# Change in Oral Impacts on Daily Performances (OIDP) with increasing age: testing the evaluative properties of the OIDP frequency inventory using prospective data from Norway and Sweden

**DOI:** 10.1186/1472-6831-14-59

**Published:** 2014-05-31

**Authors:** Ferda Gülcan, Elwalid Nasir, Gunnar Ekbäck, Sven Ordell, Anne Nordrehaug Åstrøm

**Affiliations:** 1 Department of Clinical Dentistry-Community Dentistry, Faculty of Medicine and Dentistry, University of Bergen, Bergen, Norway; 2 Örebro County Council, Örebro, Sweden; 3 School of Health and Medical Sciences, Örebro University, Örebro, Sweden; 4 Dental Commissioning Unit, Östergötland County Council, Linköping University, Linköping, Sweden

**Keywords:** Cohort, OIDP, Responsiveness, Aged, Norway, Sweden

## Abstract

**Background:**

Oral health-related quality of life, OHRQoL, among elderly is an important concern for the health and welfare policy in Norway and Sweden. The aim of the study was to assess reproducibility, longitudinal validity and responsiveness of the OIDP frequency score. Whether the temporal relationship between tooth loss and OIDP varied by country of residence was also investigated.

**Methods:**

In 2007 and 2012, all inhabitants born in 1942 in three and two counties of Norway and Sweden were invited to participate in a self-administered questionnaire survey. In Norway the response rates were 58.0% (4211/7248) and 54.5% (3733/6841) in 2007 and 2012. Corresponding figures in Sweden were 73.1% (6078/8313) and 72.2% (5697/7889), respectively.

**Results:**

Reproducibility of the OIDP in terms of intra-class correlation coefficient (ICC) was 0.73 in Norway and 0.77 in Sweden. The mean change scores for OIDP were predominantly negative among those who worsened, zero in those who did not change and positive in participants who improved change scores of the reference variables; self-reported oral health and tooth loss. General Linear Models (GLM) repeated measures revealed significant interactions between OIDP and change scores of the reference variables (p < 0.05). Stratified analysis revealed that the mean OIDP frequency score worsened in participants who became dissatisfied- and improved in participants who became satisfied with oral health. Compared to participants who maintained all teeth, those who lost teeth were more likely to experience improvement and worsening of OIDP across both countries. The two-way interaction between country and tooth loss was not statistically significant.

**Conclusions:**

Changes in OIDP at the individual level were more pronounced than the percentage distribution of OIDP at each point in time would suggest. The OIDP frequency score showed promising evaluative properties in terms of acceptable longitudinal validity, responsiveness and reproducibility among older people in Norway and Sweden. This suggests that the OIDP instrument is able to detect change in the oral health status that occurred over the 5 year period investigated. Norwegian elderly were more likely to report worsening in OIDP than their Swedish counterparts. Disease prevention should be at focus when formulating the health policy for older people.

## Background

Population ageing occurs globally and by 2050 people above 80 years will comprise 20% of the world’s population [1]. As a consequence of living longer and retaining more natural teeth, the treatment decisions for elderly patients become more complex and their need for oral health care services increasingly prominent [2]. In light of changes in the population structure and epidemiology of oral diseases, it is important to address research issues that will inform delivery of oral health care services for the elderly [2-5]. Exploring and promoting ways in which oral health care can be improved and maintained on entering old age should be encouraged. Measures of OHRQoL may play an important role by identifying needs, selecting therapies and monitoring patient progress [5-7]. OHRQoL measures have been used increasingly in oral health surveys, clinical trials and evaluations of oral health care programs. However, few investigators have examined changes in perceived oral health of older populations across socio-cultural contexts [4,6,8,9]. This is an important omission considering the many benefits of using subjective oral health indicators in clinical- and oral health care services research.

In Norway and Sweden, the availability of oral health care services among non-institutionalized, community-dwelling elderly is good. However, evidence suggests that there has been country variation regarding the accessibility to oral health care [10-12] that might be attributed to their specific organization and financing of the health care services. In both countries the financing of oral health care for adults is primarily based on patients’ payment. However, the division of labor between public and private sector, the financing of the services and the coverage of dentists differ. In Sweden, dental coverage systems were implemented for the adult population in 2008 to protect from high costs and to support oral examinations and preventive services [10-14]. A further interesting feature in Sweden is the free outreach system to actively seek out those with highest need for oral health care implemented since 1999. Although in Norway there are several social security- and welfare benefit schemes by which particular groups are refunded there is no general reimbursement of the costs of private dental care by public funds and Sweden has implemented benefit schemes for the total adult population that are of a more universal nature. Socio-cultural differences between countries regarding the provision of oral health care services to adult populations may influence dentition status and OHRQoL among the elderly.

The Oral Impacts on Daily Performance inventory (OIDP) is one of many self-reported inventories to assess OHRQoL in terms of adverse impacts that oral conditions can have on everyday life experiences [15]. The OIDP has been demonstrated to have appropriate psychometric properties when applied in population based cross-sectional surveys of elderly in Norway, Sweden, Greece and UK, just to mention a few as well as in middle- and low income countries [16-20]. Studies have shown that OIDP is associated in the expected direction with self-reported oral health and clinical indicators and that personal-, socio-demographic-, and health care service related factors modify those relationships [15,18,19,21-24]. Although OIDP has proven appropriate as a discriminative and descriptive measure in cross-sectional studies, there is less evidence on whether this inventory is suitable as an evaluative measure, to assess within individual change in oral health occurring naturally by ageing or as a consequence of interventions [8,9]. Longitudinal validity, responsiveness and ability to detect improvements and deteriorations in dentition status are necessary technical properties of an evaluative measure. Some evidence of the longitudinal validity of OHRQoL instruments generally have been provided in that substantial changes in quality of life scores have followed therapeutic regimens [9,25-28]. The longitudinal validity of the OIDP inventory and how its’ evaluative properties may be influenced by country of residence has received little attention.

Following Norwegian and Swedish cohorts of non-institutionalized elderly from age 65- to 70 years, this study assessed reproducibility, longitudinal validity and responsiveness of the OIDP frequency score within each country using change scores of satisfaction with oral health, satisfaction with tooth appearance and tooth loss as references. This study also assessed whether the temporal relationship between tooth loss and OIDP varied according to country of residence.

## Methods

### Study population

In 2007, a self-administered questionnaire initially developed in Swedish and translated into Norwegian, was mailed by Statistics Norway to all persons born in 1942 residing in the counties: Hordaland (n = 3831), Sogn and Fjordane (n = 975) and Nordland (n = 2442). These counties were chosen not only as representing rural and urban parts, but also due to known variability in oral conditions [10]. Names and addresses were obtained from public population records of Statistics Norway in April 2007. The study took place from June to August 2007 and was approved by the Ethics Committee of the Norwegian Social Science Services (NSD) (Dnr15386). The final response rate was 58.0% (n = 4211of a net population N = 7248). In September/ November 2012, the questionnaire was mailed to all persons aged 70 (born in 1942) in the three counties. The final response rate was 54.5% (n = 3733 of a net population N = 6841). Of the cohort members who completed the 2007 survey (n = 4211), a total of 70.0% (n = 2947) also participated in 2012.The 2012 survey was approved by Regional Committees for Medical and Health Research Ethics (REK) (Dnr 2012/782).

In February/April 2007, an identical questionnaire was mailed to all persons born in 1942 and residing in two counties of Sweden: Örebro (n = 3377) and Östergötland (n = 4936). The final response rate was 73.1% (n = 6078 of the net population N = 8313). This study was part of a cohort study approved by the Ethics Committee in Örebro and Östergötland when it was initiated in 1992. In March/May 2012, the total population of 70-year-olds who were invited to participate in the questionnaire survey was N = 3201 in Örebro and N = 4688 in Östergötland. The response rate was 72.2% (n = 5697 of a net population N = 7889). A total of 4862 (80.0%) participated both in 2007 and 2012. The 2007 and 2012 studies were approved by the Ethics Committee of Uppsala, Sweden (Dnr 2006/251).

### Measures

To ensure comparability of data, identical questionnaires were used and administered in the same way at each data collection in Norway and Sweden. *Socio-economic status* was assessed in terms of country of birth, marital status and education. *Self-reported oral health status* was assessed by asking “Are you generally satisfied with your teeth?” and “Are you satisfied with appearance of your teeth?” recorded on a 4-point Likert scale from (1) very satisfied to (4) not satisfied at all. The variables were dichotomized into (0) satisfied with oral health/tooth appearance (including categories 1 and 2) and (1) dissatisfied oral health/tooth appearance (including categories 3 and 4). Change scores were calculated by subtracting 2012 scores from 2007 scores and then categorized with negative mean change scores indicating worsening; zero mean change scores no change (stability) and positive mean change scores indicating improvement across time. *Dentition status (tooth loss)* was assessed by asking “How many of your own teeth do you still have (excluding baby teeth)?” The variable was categorized as (1) all (28–32 teeth), (2) missing few teeth, (3) missing quite many teeth, (4) almost no teeth left and (5) edentulous. This variable was dichotomized into (0) all or almost all teeth (including categories 1 and 2) and (1) lost many teeth (including categories 3, 4 and 5). A trajectory score of tooth loss was constructed from dummy variables in 2007 and 2012 with the categories of (0) stable all teeth, (1) tooth loss and (2) stable tooth loss. A study of validation of the question about tooth loss was performed including 26 people aged 65+ in Norway. Participants were asked the question about tooth loss and counted their own teeth. In addition a clinical oral examination was performed whereby the number of teeth was counted. Kappa value was 0.69 between counted teeth and the question of tooth loss.

A method of self-administration was applied to assess oral health-related quality of life (OHRQoL). Two previous studies have shown high level of agreement between the self-administration and interview method administered Child-OIDP [29,30]. OHRQoL was assessed using the eight-item “Oral Impacts on Daily Performance” (OIDP) frequency inventory. “During the past 6 months, how often have problems with your mouth and teeth caused you any difficulty with: eating and enjoying food; speaking and pronouncing clearly: cleaning teeth; sleeping and relaxing; smiling and showing teeth without embarrassment; maintaining usual emotional state; enjoying contact with people and carrying out major work?” Each item was scored on a 5-point scale, as follows: (1) never affected, (2) less than once a month, (3) once or twice a month, (4) once or twice a week, (5) every/ nearly every day. For the purpose of analysis the items were dichotomized into (1) affected (including categories 2–5) and (0) never affected (the category 1). Sum scores OIDP frequency ADD (8–40) and OIDP frequency SC (0–8) were computed by adding the 8 performance scores as originally scored and the dichotomized performance scores, respectively. OIDP frequency SC score was dichotomized into (0) no daily performance affected (including score 0) and (1) at least one daily performance affected (including score 1 to 8). Change scores for the OIDP frequency ADD scores and the sub-scale scores were constructed by subtracting the 2012 from the 2007 scores. A positive mean change score indicated improvement, a negative mean change score indicated worsening and zero indicated stability or no change [7]. Minimal important difference (MID) or the smallest score of change considered important from the patients’ and clinicians’ point of view were calculated using the distribution based approach. Effect sizes were calculated by dividing the mean OIDP change scores by the standard deviation of the corresponding baseline scores [25].

### Statistical analysis

Data were analyzed using Statistical Package for Social Sciences 20 (SPSS Inc., Chicago, IL, USA). Internal consistency reliability was evaluated using Cronbach’s alpha. Changes in prevalence of any impacts in OIDP and subscale scores were assessed using Cochrane’s Q. Test-retest reliability was assessed using the intra-class correlation coefficient (ICC). Longitudinal validity was calculated by evaluating the association between OIDP change scores and categorical reference variables using One-Way ANOVA and Bonferroni post hoc test. General Linear Models (GLM) for repeated measures were used to assess the within individual change of OIDP ADD scores by categorical reference variables. Within group changes were assessed using Wilcoxon Matched pair signed test. To assess the independent contribution of categorical reference variables to change in OIDP, multiple variable logistic regression analysis was performed with odd ratios (OR) and 95% confidence interval (CI) using worsening and improvements of OIDP as dependent variables (worsening versus all others and improved versus all others) and change scores of reference variables as independents, adjusting for sex and country of residence. Two-way interactions between country and reference variables upon OIDP were tested.

## Results

### Socio-demographic distribution and loss to follow-up

In Norway, there were statistically significant differences between the groups who were and were not successfully followed-up with respect to; satisfaction with oral health, satisfaction with tooth appearance, tooth loss and OIDP (Table 1). The 2947 Norwegian cohort members included in the analyses consisted of 48.8% women. Totals of 86.5% and 98.1% were married and native born in Norway, respectively. In Sweden, there were statistically significant differences between responders and non-responders with respect to socio-demographics, OIDP and the reference variables as measured at baseline (Table 1). The 4862 cohort members included in the analyses consisted of 51.2% women. Moreover, totals of 94.6% and 79.2% were native born and married in Sweden, respectively.

**Table 1:** Socio-demographics and oral health status at baseline according to follow- up status in Norway and Sweden

	**Norway**			**Sweden**		
	**Lost to follow-up n = 1264 % (n)**	**Followed up n = 2947 % (n)**	**Baseline n = 4211 % (n)**	**Lost to follow-up n = 1216 % (n)**	**Followed up n = 4862 % (n)**	**Baseline n = 6078 % (n)**
*Gender*						
Males	48.3(561)	51.2(1486)	50.4(2047)	51.4(625)	48.8(2373)	49.3(2998)
Females	51.7(600)	48.8(1415)	49.6(2015)	48.6(591)	51.2(2489)	50.7(3080)
*Marital status*						
Unmarried	15.5(162)	13.5(362)	14.1(524)	32.2(378)	20.8(995)	23.1(1373)
Married	84.5(883)	86.5(2314)	85.9(3197)	67.8(797)	79.2(3781)**	76.9(4578)
*Country of birth*						
Native	97.2(1120)	98.1(2822)	97.8(3942)	90.1(1057)	94.6(4520)**	93.7(5577)
Foreign	2.8 (32)	1.9 (56)	2.2 (88)	9.9 (116)	5.4 (259)	6.3 (375)
*OIDP*						
OIDP = 0	66.7(724)	71.0(1975)	69.8(2699)	67.2(751)	72.7(3375)	71.6(4126)
OIDP > 0	33.3(361)	29.0(806)*	30.2(1167)	32.8(367)	27.3(1269)**	28.4(1636)
*Satisfaction with oral health*						
Satisfied	72.2(824)	78.2(2241)**	76.5(3065)	69.2(801)	78.6(3748)**	76.8(4549)
Dissatisfied	27.8(318)	21.8(625)	23.5(943)	30.8(356)	21.4(1018)	23.2(1374)
*Satisfaction with tooth appearance*						
Satisfied	75.4(859)	80.3(2306)**	78.9(3165)	72.2(837)	78.9(3768)**	77.6(4605)
Dissatisfied	24.6(280)	19.7(567)	21.1(847)	27.8(323)	21.1(1008)	22.4(1331)
*Tooth loss*						
All/Almost all teeth	65.4(738)	78.2(2224)	74.6(2962)	62.8(723)	74.1(3515)	71.9(4238)
Lost teeth	34.6(390)	21.8(619)**	25.4(1009)	37.2(428)	25.9(1230)**	28.1(1658)

Chi Square test: *p < 0.05, **p < 0.001.

### Change in prevalence of OIDP and reference variables

According to Table 2, the prevalence of OIDP frequency score in Norway was 29.0% and 28.4% (n.s) in 2007 and 2012, respectively. Corresponding figures for the subscale OIDP frequency scores ranged from 21.1% versus 20.9% (eating) to 3.9% versus 3.5% (work relations) (n.s). The mean OIDP frequency ADD scores in 2007 and 2012 were 9.5 (sd = 3.9) and 9.4 (sd = 3.8) (n.s) (not in table). At both survey occasions, eating and smiling were the impacts most frequently reported (Table 2). In Sweden, the mean OIDP ADD score declined from 9.7 (sd = 4.5) to 9.0 (sd = 3.4) (p < 0.001) (not in table), whereas the prevalence of impacts declined from 27.3% in 2007 to 20.4% in 2012 (p < 0.001) (Table 2). The prevalence of OIDP subscale scores ranged from 19.0% versus 15.5% (eating) to 2.9% versus 2.0% (work relations) (p < 0.001). In 2007, eating and emotions were the impacts most frequently reported. Corresponding figures in 2012 were eating and smiling. The prevalence of tooth loss increased from 21.8% to 23.2% in Norway and from 25.9% to 27.3% in Sweden (p < 0.001) (Table 2).

**Table 2:** **Prevalence% (n) of any impacts in OIDP and subscale scores, dissatisfaction with oral health, dissatisfaction with tooth appearance and tooth loss in 2007 and 2012 (The Norwegian cohort n = 2947) (The Swedish cohort n = 4862**)

	**Norway**		**Sweden**	
	**2007**	**2012**	**2007**	**2012**
OIDP	29.0 (806)	28.4 (796)ns	27.3 (1269)	20.4 (935)**
Eating	21.1 (601)	20.9 (604)ns	19.0 (900)	15.5 (728)**
Speaking	7.8 (223)	8.4 (243)ns	5.2 (245)	4.7 (223)ns
Cleaning	11.7 (332)	11.8 (341)ns	8.0 (380)	6.1 (286)**
Sleeping	7.4 (211)	7.0 (203)ns	7.7 (363)	6.1 (287)**
Smiling	12.6 (357)	11.7 (333)ns	9.5 (448)	6.9 (326)**
Emotion	8.1 (229)	8.3 (235)ns	15.5 (732)	6.2 (290)**
Social	8.7 (247)	8.2 (234)ns	8.7 (410)	5.5 (257)**
Work	3.9 (111)	3.5 (99)ns	2.9 (139)	2.0 (94)*
Dissatisfaction with oral health	21.8 (625)	18.8 (542)**	21.4 (1018)	17.9 (850)**
Dissatisfaction with tooth appearance	19.7 (567)	16.8 (485)**	21.1 (1008)	17.4 (829)**
Lost teeth	21.8 (619)	23.2 (655)**	25.9 (1230)	27.3 (1276)**

Cochrane’s Q test: *p < 0.05, **p < 0.001, ns- not statistically significant.

### Change scores, longitudinal validity and responsiveness

In Norway, 71.8%, 11.8% and 16.3% reported no change, worsening and improvement, regarding satisfaction with oral health. Corresponding figures for satisfaction with tooth appearance were 73.8%, 10.6% and 15.3%. Totals of 76.0% remained in the category having almost all teeth, 5.5% experienced tooth loss and 18.5% were stable with respect to reporting major tooth loss across the survey years. The majority of subjects who reported no change in the reference variable were reflected by the OIDP change scores. Totals of 63.6%, 17.7% and 18.7% reported no change, worsening and improvement, respectively. In Sweden, totals of 70.3%, 12.8% and 16.9% reported no change, worsening and improvement with respect to satisfaction with oral health. The corresponding rates for satisfaction with tooth appearance were 70.8%, 12.3% and 17.0%. Totals of 71.5%, 7.3% and 21.1% were stable with reporting all teeth, experienced tooth loss and were stable with reporting having major tooth loss. Totals of 68.1%, 11.5% 20.4% reported no change, worsening and improvement regarding OIDP scores (not shown in table).

Table 3 depicts the mean change OIDP scores by change scores in the categorical reference variables. Within each country, mean OIDP change scores (and the mean OIDP change subscale scores not shown in table) were negative (worsened) among those who reported worsened satisfaction with oral health and tooth appearance, about zero in subjects who were stable and positive (improved) in subjects reporting improvements in satisfaction with oral health and tooth appearance. Moreover, mean OIDP change score (and the mean OIDP change subscale scores) were about zero for those who maintained almost all teeth and were stable with respect to reporting major tooth loss and negative with those who reported tooth loss between 2007 and 2012. Statistically significant gradients (p < 0.001) were observed according to all reference variables in both countries. Responsiveness was estimated by calculating effect sizes for the distribution of OIDP change scores according to the reference variables. In Norway and Sweden the effect sizes ranged from 0.0 to 0.5 and from 0.1 to 0.4, respectively (Table 3).

**Table 3:** Longitudinal validity: mean change OIDP scores (sd) and [effect sizes] by change scores of reference variables (Norwegian cohort n = 2947) (Swedish cohort n = 4862)

	**Satisfaction with oral health**			
	**Worsened**^**a**^	**Stable**^**b**^	**Improved**^**c**^	**Total**
*Norway*				
OIDP change score	−1.60 (5.2) [0.4]	0.02 (2.3)[0.0]	1.28 (4.3)[0.2]**	0.04 (3.3) [0.1]
*Sweden*				
OIDP change score	−0.56 (4.1) [0.2]	0.28 (2.7) [0.1]	2.14 (5.4) [0.4]**	0.49 (3.6) [0.1]
	**Satisfaction with tooth appearance**			
	**Worsened**^**a**^	**Stable**^**b**^	**Improved**^**c**^	**Total**
*Norway*				
OIDP change score	−1.93 (5.4) [0.5]	0.03 (2.5)[0.0]	1.41 (3.9)[0.3]**	-
*Sweden*				
OIDP change score	−0.59 (4.0)[0.2]	0.34 (2.9)[0.1]	1.88 (5.3) [0.3]**	-
	**Tooth loss**			
	**Lost teeth**^**a**^	**Stable all teeth**^**b**^	**Stable tooth loss**^**c**^	**Total**
*Norway*				
OIDP change score	−0.72 (4.6)[0.4]	0.01 (2.0)[0.0]	0.21 (6.1)[0.3]*	-
*Sweden*				-
OIDP change score	−0.14 (3.9) [0.4]	0.25 (2.1)[0.1]	1.13 (6.4) [0.2]**	-

Data are given as mean (sd) [effect size].

One-way ANOVA: *p < 0.05, **p < 0.001.

Bonferroni post hoc analyses indicated the following (p < 0.05):

● Statistically significant differences in mean OIDP change by change score of satisfaction with oral health and change score of satisfaction with tooth appearance in *Norway* and *Sweden*: group a vs. group b, group a vs. group c and group b vs. group c.

● Statistically significant differences in mean OIDP change by change score of tooth loss in *Norway*: group a vs. group b, group a vs. group c.

● Statistically significant differences in mean OIDP change by change score of tooth loss in *Sweden*: group a vs. group c and group b vs. group c.

GLM repeated measures revealed statistically significant interactions between OIDP scores and change scores of categorical reference variables in both countries (Table 4). In Norway, statistically significant interactions occurred between OIDP scores and change scores of satisfaction with oral health (Wilk’s λ = 0.946, p < 0.001), satisfaction with tooth appearance (Wilk’s λ = 0.935, p < 0.001) and tooth loss (Wilk’s λ = 0.997, p < 0.05). Estimated marginal means ranged from 9.9 (sd = 4.3) to 11.5 (sd = 5.8) and from 10.8 (sd = 5.4) to 9.5 (sd = 3.9) within the groups who worsened and improved their satisfaction with oral health and from 9.8 (sd = 3.6) to 10.6 (sd = 4.4) in those who reported tooth loss (Table 4). In Sweden, statistically significant interactions occurred between OIDP scores and change scores of satisfaction with oral health (Wilk’s λ = 0.952, p < 0.001), satisfaction with tooth appearance (Wilk’s λ = 0.963, p < 0.001) and tooth loss (Wilk’s λ = 0.988, p < 0.001). The estimated marginal means ranged from 9.4 (sd = 3.9) to 9.9 (sd = 4.5) and from 10.9 (sd = 5.8) to 8.8 (sd = 2.9) within the groups who worsened and improved their satisfaction with oral health and from 9.4 (sd = 3.3) to 9.5 (sd = 3.7) in those who experienced tooth loss (Table 4).

**Table 4:** Responsiveness of OIDP: mean OIDP in 2007 and 2012 by change scores of reference variables in Norway (n = 2947) and Sweden (n = 4862)

**Satisfaction with oral health**	***Worsened Mean (sd)***^***c***^	***Stable Mean (sd)***^***d***^	***Improved Mean (sd)***^***e***^	***Wilk’s lamda p-value***
**Norway**				
OIDP 2007	9.9 (4.3)	8.9 (3.1)	10.8 (5.4)	
OIDP 2012	11.5 (5.8)	8.9 (3.2)	9.5 (3.9)	
2007 versus 2012	p = 0.001^a^	p = 0.792^a^	p = 0.001^a^	0.946 p = 0.001^b^
**Sweden**				
OIDP 2007	9.4 (3.9)	9.2 (3.7)	10.9 (5.8)	
OIDP 2012	9.9 (4.5)	8.8 (3.0)	8.8 (2.9)	
2007 versus 2012	p = 0.001^a^	p = 0.000^a^	p = 0.000^a^	0.952 p = 0.001^b^
**Satisfaction with tooth appearance**	***Worsened Mean (sd)***^***c***^	***Stable Mean (sd)***^***d***^	***Improved Mean (sd)***^***e***^	
**Norway**				
OIDP 2007	9.7 (3.9)	9.1 (3.4)	10.7 (5.1)	
OIDP 2012	11.6 (6.0)	9.1 (3.4)	9.3 (3.1)	
2007 versus 2012	p = 0.001^a^	p = 0.987^a^	p = 0.001^a^	0.935 p = 0.001^b^
**Sweden**				
OIDP 2007	9.2 (3.5)	9.2 (3.6)	10.8 (5.6)	
OIDP 2012	9.8 (4.6)	8.8 (2.9)	8.9 (3.1)	
2007 versus 2012	p = 0.002^a^	p = 0.000^a^	p = 0.000^a^	0.963 p = 0.001^b^
**Tooth loss**	***Lost teeth Mean (sd)***^***c***^	***Stable all teeth Mean (sd)***^***d***^	***Stable tooth loss Mean (sd)***^***e***^	
**Norway**				
OIDP 2007	9.8 (3.6)	8.5 (1.8)	12.8 (6.8)	
OIDP 2012	10.6 (4.4)	8.5 (1.7)	12.6 (6.9)	
2007 versus 2012	p = 0.032^a^	p = 0.916^a^	p = 0.243^a^	0.997 p = 0.05^b^
**Sweden**				
OIDP 2007	9.4 (3.3)	8.6 (2.0)	12.1 (6.9)	
OIDP 2012	9.5 (3.7)	8.3 (1.3)	10.9 (5.9)	
2007 versus 2012	p = 0.690^a^	p = 0.000^a^	p = 0.000^a^	0.988 p = 0.001^b^

^a^Wilcoxon matched pair signed rank test.

^b^GLM repeated measure.

^c^Bonferroni post hoc analyses indicated the following (p < 0.05):

● Statistically significant differences in mean OIDP change by change score of satisfaction with oral health, satisfaction with tooth appearance and tooth loss in *Norway*: group c vs. group d, group c vs. group e and group d vs. group e.

● Statistically significant differences in mean OIDP change by change score of satisfaction with oral health and satisfaction with tooth appearance in *Sweden*: group c vs. group d and group d vs. group e.

● Statistically significant differences in mean OIDP change by change score of tooth loss in *Sweden*: group c vs. group d, group c vs. group e and group d vs. group e.

### Reliability

Internal consistency reliability of OIDP in terms of Cronbach’s alpha in 2007 and 2012 were 0.89 in both countries. In Norway, the 1723 subjects who reported no change in satisfaction with oral health were used to assess test-retest reliability of the total OIDP score [8]. The intra-class correlation coefficient was 0.73 (95% CI 0.70-0.75). Corresponding figures in Sweden among 3294 who reported no change in satisfaction with oral health was 0.77 (95% CI 0.75-0.78) (not shown in table).

### Country variation in responsiveness to change

According to Table 5, multiple variable logistic regression analyses revealed that worsening of OIDP was less likely in Sweden than in Norway. All categorical reference variables contributed to the improvement and worsened of OIDP across the two countries, with change in tooth loss being the strongest covariate. Compared to subjects who maintained almost all teeth, subjects who lost teeth and were stable with reporting tooth loss across time were more likely to experience worsening in OIDP. The corresponding ORs were 3.3 (95% CI 2.6-4.2) and 3.5 (95% CI 2.9-4.2). Likewise, tooth loss was the strongest covariate of improvement in OIDP after adjusting for country- and other categorical reference variables. Compared to those who maintained all teeth, those who reported tooth loss both in 2007 and 2012 were more likely to report improved OIDP. The corresponding ORs were 1.7 (95% CI 1.3-2.1) and 3.2 (95% CI 2.8-3.8). No two-way interactions between country and tooth loss upon worsening and improvement in OIDP were statistically significant, suggesting that the responsiveness to change of the OIDP inventory did not vary between countries.

**Table 5:** Worsened and improved OIDP from 2007 to 2012 (improved versus all others and worsened versus all others) regressed on country of residence and change scores of reference variables, OR and 95% CI

	**Worsened OIDP**		**Improved OIDP**	
	**Unadjusted OR (95% CI)**	**Adjusted OR (95% CI)**	**Unadjusted OR (95% CI)**	**Adjusted OR (95% CI)**
Norway	1	1	1	1
Sweden	0.6 (0.5-0.7)	0.6 (0.5-0.6)	1.1 (0.9-1.3)	1.1 (0.9-1.2)
**Satisfaction with oral health**				
Stable	1	1	1	1
Worsened	2.9 (2.5-3.5)	1.9 (1.6-2.3)	0.9 (0.7-1.1)	0.8 (0.6-1.0)
Improved	0.6 (0.5-0.8)	0.5 (0.4-0.7)	3.3 (2.8-3.8)	2.0 (1.7-2.4)
**Satisfaction with tooth appearance**				
Stable	1	1	1	1
Worsened	2.6 (2.2-3.1)	1.8 (1.5-2.2)	0.9 (0.7-1.0)	0.8 (0.7-1.1)
Improved	0.6 (0.5-0.8)	0.6 (0.5-0.8)	2.8 (2.4-3.2)	1.8 (1.5-2.1)
**Change tooth loss**				
Stable all teeth	1	1	1	1
Lost teeth 2007-2012	3.6 (2.9-4.6)	3.3 (2.6-4.2)	1.5 (1.2-2.0)	1.7 (1.3-2.1)
Stable tooth loss	3.2 (2.7-3.7)	3.5 (2.9-4.2)	3.6 (3.1-4.2)	3.2 (2.8-3.8)
Nagelkerke’s R^2^	13.5			12.4

## Discussion

This study presents one of very few undertaken to assess the evaluative properties of the OIDP frequency inventory, focusing non-institutionalized elderly in Norway and Sweden. Moreover, this study assessed the magnitude and direction of change in the OIDP frequency inventory to further understand the development of older peoples’ OHRQoL by increasing age. The cross-sectional validity of the OIDP has been assessed previously in national samples of adults in Norway and Sweden [16,17]. According to Locker & Jakovic [6], Locker [7] and Locker & Jakovic [8], both cross-sectional and longitudinal psychometric properties of an OHRQoL inventory should ideally be established in every sample and context under consideration. Important steps in the process of psychometric evaluation of the OIDP are tests of its internal consistency reliability and reproducibility. In this study, Cronbach’s alpha amounted to 0.89 at both measurement occasions and in both countries. This is above the recommended values of 0.70 and consistent with those previously reported in surveys of older people [15,18,19,21,23]. Reproducibility amounted to 0.73 and 0.77 in Norway and Sweden, indicating good stability at both sites [18]. However, reproducibility alone does not guarantee satisfactory evaluative properties. The main purpose of the study was to assess the longitudinal validity and responsiveness of the OIDP that is whether or not this inventory is responsive to changes in oral health occurring naturally or as a consequence of intervention. Without this evidence, it cannot be ascertained whether any change in OIDP represents real change or measurement error. The mean OIDP change scores translated into effect sizes (estimations of minimal important differences, MID) ranging from 0.1 to 0.5 showed a clear gradients across the change groups of the reference variables (Table 3). The effect sizes indicated small to moderate magnitude of change using Cohen’s Benchmarks [31]. A value of 0.2 should be considered small, a value of 0.4 moderate and a value of 0.8 and above large effect [31].

It should be noted that the change scores presented could be confounded by regression towards the mean effect. Thus, those with more extreme scores at baseline tended to have less extreme scores at follow-up regardless of any real change in the characteristics being measured. Moreover, the great floor effect of the OIDP frequency score (prevalence of no impacts) may have limited its sensitivity to change at the extremes of oral health [25]. Whether the small to moderate changes presented here are clinically meaningful, what specific clinical conditions account for the changes remains important topic for further research. In this study and for the purpose of assessing longitudinal validity, evaluation of OIDP was limited to the comparison with change scores of self-reported oral health and tooth loss.

Results from the multivariable logistic regression analysis confirmed by and large those based on bivariate analysis. Taken together favorable and unfavorable changes in the reference variables across time were reflected by improvement and deterioration of the OIDP frequency scores. This finding has support in previous studies of prospective design [6,8,32]. Tooth loss emerged as a strong covariate of oral impacts across time independent of residence country. Accordingly, a recent systematic review of observational studies revealed that tooth loss associated with worsened OHRQoL across socio-cultural contexts and independent of the specific OHRQoL measure utilized [33]. However, participants who reported tooth loss at both survey occasions were about three times more likely to experience worsened and improved OIDP across time. Focusing elderly in Brazil, de Andrade [9] reported number of missing teeth at baseline to be the best predictor of both improvement and deterioration of OHRQoL scores at five years follow-up. There is also evidence that high risk groups (stability in reported major tooth loss) are more likely to experience both deterioration and improvement in OHRQoL compared with low risk groups and that the positive relationship between tooth loss and worsened OHRQoL is not a simple monotonic one [4,34]. For some people, tooth loss might lead to pain relief and improved OHRQoL, whereas others may experience chewing difficulties, impaired function and problems with prosthesis leading to deteriorated OHRQoL [6,8].

Among the strengths of this study is the use of a cross-cultural prospective cohort design recognized to be highly relevant when measuring change in oral health status [7]. Although the response rate to the follow-up was good in both countries, those who completed the survey at age 70 had better oral health at age 65 than those who were lost to follow-up. Thus, the two groups differed on variables that associated with change in OIDP, implying that the generalization of the results presented should be made with caution. Due to possible selection bias, the worsening of OIDP across the survey period might be an underestimate of that actually occurring in the total sample, particularly in Norway with highest rate of non-response. However, even in those relatively well educated cohorts investigated (about one third having university education in both countries), 20%- 30% reported oral impacts and dissatisfaction with oral health suggesting need for oral health care and treatment. A second strength of this study was use of different methods to assess change, as recommended by Locker [7]. In accordance with the present results, previous studies, also from Norway, have shown positive associations between age and tooth loss and negative associations between age and OHRQoL, respectively. However, due to their cross-sectional design, the temporal relationships have been unclear [23,35]. Longitudinal studies conducted elsewhere have reported on non-significant change in OHRQoL with increasing age in older populations [35]. In both countries about half of the participating subjects reported no change in OIDP scores, whereas about one fourth reported increase and decline during the five year survey period. This is consistent with findings among older Canadians, using the global transition scores whereby the majority reported no change across a 3-year survey period [7].

Whereas some intervention studies have addressed the evaluative properties of the OIDP inventory, this study adds to the literature by demonstrating its responsiveness to change in oral health occurring naturally among non-institutionalized elderly in a cross-cultural context [25-27]. Thus, as a longitudinal cohort study without the inclusion of an intervention of known efficacy, the changes observed might document the natural history of changes in oral health of elderly in Norway and Sweden between age 65 and 70. According to the present results, the responsiveness of OIDP to changes in tooth loss or the influence of changes in tooth loss on changes in OIDP was not dependent on study site. On the other hand, Swedish participants were less likely than their Norwegian counterparts to experience impaired OIDP. This indicates influence from a cultural dimension on the development of OHRQoL across time in older persons as suggested by previous studies [6,19]. Alternatively, this variation may be attributed to differences between Norway and Sweden regarding structure and financing of oral health care systems, such as the implementation of benefit schemes that in Sweden are of a more universal nature and the fact that per capita spending on oral health and the rate of regular adult dental attendance have been higher in Sweden than in Norway [10-12]. Previous evidence, suggesting that access to dental care acts as a proxy for OHRQoL, gives resonance here [2,23].

## Conclusion

Changes in OIDP at the individual level were more pronounced than the percentage distribution of OIDP at each point in time would suggest. The OIDP frequency score showed promising evaluative properties in terms of acceptable longitudinal validity, responsiveness and reproducibility among older people in Norway and Sweden. This suggests that the OIDP frequency instrument is able to detect change in the oral health status that occurred over the 5 year period investigated. Norwegian elderly were more likely to report worsening in OIDP than their Swedish counterparts. Disease prevention should be at focus when formulating the health policy for older people.

## Competing interests

The authors declare that they have no competing interests.

## Authors’ contributions

FG: carried out data analysis and contributed to manuscript writing. EN: contributed data analysis. GE and SO: provided data from Sweden and revised manuscript writing. ANÅ: conceived of the study in Norway, supervised data analysis and contributed to manuscript writing. All authors read and approved the final manuscript.

## Pre-publication history

The pre-publication history for this paper can be accessed here:

http://www.biomedcentral.com/1472-6831/14/59/prepub
