# Erratum to: Change in Oral Impacts on Daily Performances (OIDP) with increasing age: testing the evaluative properties of the OIDP frequency inventory using prospective data from Norway and Sweden

**DOI:** 10.1186/s12903-015-0043-5

**Published:** 2015-05-12

**Authors:** Ferda Gülcan, Elwalid Nasir, Gunnar Ekbäck, Sven Ordell, Anne Nordrehaug Åstrøm

**Affiliations:** Department of Clinical Dentistry-Community Dentistry, Faculty of Medicine and Dentistry, University of Bergen, Bergen, Norway; Örebro County Council, Örebro, Sweden; School of Health and Medical Sciences, Örebro University, Örebro, Sweden; Dental Commissioning Unit, Östergötland County Council, Linköping University, Linköping, Sweden

## Erratum

After the publication of this work [1], we became aware that the total number of being “unmarried” in Table 1 was incorrect. In the Norwegian study, marital status was originally categorized as (1) married, (2) unmarried, (3) divorced and (4) widowed. For the purpose of analysis, the variable was dichotomized as (1) married (including the original category 1) and (0) unmarried (including the original categories (2,3), whereas the original category (4) was set to missing instead of correctly being included into the unmarried category of the dichotomy variable. This error has been corrected in a revised Table 1 included in this erratum. The new categorization of the variable marital status did not influence the results and conclusion of the article. We regret any inconvenience that this inaccuracy might have caused.

**Table 1:** Socio-demographics and oral health status at baseline according to follow-up status in Norway and Sweden

	**Norway**			**Sweden**		
	**Lost to**			**Lost to**		
	**Follow-up**	**Followed up**	**Baseline**	**Follow-up**	**Followed up**	**Baseline**
	**n = 1264**	**n = 2947**	**n = 4211**	**n = 1216**	**n = 4862**	**n = 6078**
	**% (n)**	**% (n)**	**% (n)**	**% (n)**	**% (n)**	**% (n)**
*Gender*						
Males	48.3(561)	51.2(1486)	50.4(2047)	51.4(625)	48.8(2373)	49.3(2998)
Females	51.7(600)	48.8(1415)	49.6(2015)	48.6(591)	51.2(2489)	50.7(3080)
*Marital status*						
Unmarried	22.9(262)	19.6(565)	20.5(827)	32.2(378)	20.8(995)	23.1(1373)
Married	77.1(883)	80.4(2314)*	79.5(3197)	67.8(797)	79.2(3781)**	76.9(4578)
*Country of birth*						
Native	97.2(1120)	98.1(2822)	97.8(3942)	90.1(1057)	94.6(4520)**	93.7(5577)
Foreign	2.8(32)	1.9(56)	2.2(88)	9.9(116)	5.4(259)	6.3(375)
*OIDP*						
OIDP = 0	66.7(724)	71.0(1975)	69.8(2699)	67.2(751)	72.7(3375)	71.6(4126)
OIDP > 0	33.3(361)	29.0(806)*	30.2(1167)	32.8(367)	27.3(1269)**	28.4(1636)
*Satisfaction with oral health*						
Satisfied	72.2(824)	78.2(2241)**	76.5(3065)	69.2(801)	78.6(3748)**	76.8(4549)
Dissatisfied	27.8(318)	21.8(625)	23.5(943)	30.8(356)	21.4(1018)	23.2(1374)
*Satisfaction with tooth appearance*						
Satisfied	75.4(859)	80.3(2306)**	78.9(3165)	72.2(837)	78.9(3768)**	77.6(4605)
Dissatisfied	24.6(280)	19.7(567)	21.1(847)	27.8(323)	21.1(1008)	22.4(1331)
*Tooth loss*						
All/Almost all teeth	65.4(738)	78.2(2224)	74.6(2962)	62.8(723)	74.1(3515)	71.9(4238)
Lost teeth	34.6(390)	21.8(619)**	25.4(1009)	37.2(428)	25.9(1230)**	28.1(1658)

Chi Square test: *p < 0.05, **p < 0.001.
